# Interdependence, marginalisation, and self-acceptance: lived experiences of cancer patients in rural China

**DOI:** 10.1080/17482631.2025.2581517

**Published:** 2025-11-09

**Authors:** Jingni Ma, Haoke Li, Mengya Zhao, Chuoyan Liang, Siqi Li, Chen Qu

**Affiliations:** a Department of Psychology, College of Liberal Arts, Wenzhou-Kean University, Wenzhou, Zhejiang, People's Republic of China; b School of Health in Social Science, The University of Edinburgh, Scotland, UK; c Department of Primary Care and Mental Health, Institute of Population Health, University of Liverpool, Liverpool, UK; d Department of Psychology, South China Normal University, Guangzhou, Guangdong, People's Republic of China

**Keywords:** Cancer patients, lived experience, interpretive phenomenological analysis, social ecological model, self-construal theory, health psychology

## Abstract

**Purpose:**

This study explores the lived experiences of cancer patients in deprived areas of China, focusing on how they understand their illness, their challenges, and their coping mechanisms through the lens of the Social Ecological Model and Self-construal Theory.

**Methods:**

A qualitative research design was employed, using semi-structured interviews and Interpretive Phenomenological Analysis (IPA) to explore these patients' experiences.

**Results:**

Twelve cancer patients (75% female) participated in this study. Patients were aged 27 to 59 years old, with an average of 12 months after diagnosis. The findings reveal a multi-level interplay of challenges and coping strategies. At the individual level, patients navigated profound financial burdens and identity changes, with self-acceptance emerging as a central coping mechanism. At the interpersonal level, relational interdependence exhibited a dual nature, experienced as both a significant source of burden and a powerful catalyst for empowerment. At the community and policy level, patients contended with systemic marginalisation within a strained healthcare system, exacerbated by inadequate health policies.

**Conclusions:**

The findings highlight the need for approaches that integrate socio-cultural and economic realities to explore the lived experiences of marginalised clinical populations comprehensively.

## 1. Introduction

Cancer patients often suffer from multifaceted challenges, such as physical discomforts (Henson et al., 2020; Navari, 2020) and psychological distress (Brierley et al., 2019; Burgess et al., 2005; Chochinov, 2002; Cook et al., 2018), and thereby also face difficulties in accessing healthcare resources and receiving financial and psychosocial support, particularly for those in deprived areas and of low socio-economic status (Asseffa, 2017; Carrera et al., 2018; Manser & Bauerfeind, 2014; Salsman et al., 2019; Song et al., 2019). Those deprived cancer patients, however, find it more difficult to cope with cancer-related sufferings and social reintegration compared to urban and affluent counterparts, because of the intensified feelings of inequalities, financial burdens, and social deprivation, the perceived loss of dignity (Chochinov, 2002; Guo & Jacelon, 2014; Xiao et al., 2020).

There are rising concerns about the socioeconomic disparities in healthcare use and financial burden among Chinese cancer patients (Zhao et al., 2022). These disparities are not unique to cancer care but are deeply embedded within China's broader healthcare landscape, which is characterized by a significant urban-rural divide. For decades, rural and deprived areas have contended with systemic challenges, including a shortage of qualified health professionals, outdated medical facilities, and lower levels of health literacy and awareness among the population (Li et al., 2020; Song et al., 2019). While national health insurance schemes like the New Rural Cooperative Medical Scheme have expanded coverage, rural residents often face lower reimbursement rates and limited access to advanced treatments, leading to disproportionately high out-of-pocket expenditures (Zhao et al., 2020). This environment of systemic scarcity and financial precarity creates a uniquely challenging context for individuals diagnosed with a catastrophic illness like cancer.

The lived experience of cancer for patients in deprived areas is profoundly influenced by a complex interplay of personal psychology, interpersonal relationships, community-level resource availability, and broader societal policies. Understanding this multifaceted reality requires a framework that can simultaneously analyze these interconnected layers. For this reason, the current study utilized the Social Ecological Model of Health (SEM, Bronfenbrenner, 1977; Wallerstein & Duran, 2003; World Health Organization, 1974) as its overarching framework. The SEM is a robust model for understanding the multifaceted influences on health, positing that an individual’s well-being is shaped by the interplay of personal, interpersonal, community, and societal factors.

To understand the cultural context of these interactions in China, Self-Construal Theory (SCT; Cross et al., 2020; Markus et al., 1991) was also integrated into our approach. SCT underscored the relationships between social environment and individuals’ self-perception (Markus et al., 1991). The theory explains the relationships via self-construals, which are broadly categorized into two types: independent and interdependent. In Western cultures, individuals with independent self-construal emphasize personal autonomy, individual rights, and self-expression. While in many Asian cultures, including China, an interdependent self-construal is more prevalent, where individuals see themselves as part of a larger social environment, prioritizing group harmony, relational obligations, and collective well-being (for a review see, Cross et al., 2020). Therefore, in China, the interpersonal and community factors may play a more important role in cancer patients’ lived experiences. For cancer patients in deprived areas, this interdependence may manifest in several ways, including the relational burdens they feel towards their families, the social isolation stemming from internalized stigma, and their coping strategies that often involve communal support and familial obligations (Su et al., 2022; Zhao et al., 2020). Thus, the predominant interdependent self-construal suggests that cancer patients often consider their illness not just as a personal battle but as one that impacts their family and community.

Despite the rising cancer prevalence and long-standing cancer treatment disparities (Wang & Jiao, 2016), the existing literature has not fully explored how these systemic and cultural factors intersect. While prior studies on Chinese cancer patients have examined financial hardship or coping strategies in isolation (Su et al., 2022; Xiao et al., 2020; Zhu et al., 2020), a key theoretical tension has been overlooked: how the cultural value of relational interdependence (explained by SCT) interacts with the structural reality of socioeconomic deprivation (mapped by SEM). For instance, it remains unclear how patients navigate the dual experiences of family as both a crucial source of support and a significant emotional burden.

Therefore, this study aims to address this gap, guided by the following central research question: How do cancer patients in deprived areas of China make sense of their illness, and what challenges do they face and coping strategies do they employ within their socio-cultural context?

## 2. Methodology

### 2.1 Participants

Twelve cancer patients (*M* = 49.33, *SD* = 12.59) were interviewed. This sample size was chosen to align with the methodological recommendations for IPA. Leading proponents of IPA suggest that a small, homogenous sample is ideal for achieving the rich, in-depth, and idiographic analysis that is the core aim of the methodology (Smith & Fieldsend, 2021). A smaller sample allows the researcher to engage deeply with the nuances and complexities of each individual's lived experience, which would be compromised with a larger group. Our sample size of twelve was deemed sufficient to identify recurrent and divergent themes while remaining manageable for the intensive, case-by-case interpretative work that IPA demands (Smith & Fieldsend, 2021).

Patients' demographic and cancer characteristics are displayed in Table I. Purposive sampling was employed in this study to recruit eligible patients. Patients were selected based on age (above 18 years old), cancer diagnosis, living with low SES (residents in rural areas and low-income residents receiving minimum living standard subsidies) and geographical location - Gansu Province is characterized by the lowest annual Gross Domestic Product (GDP) among the provinces in mainland China (Ma et al., 2019), thereby signifying its status as a comparatively less economically developed region within the national framework.

**Table I.** Cancer Patients' demographic information.

Patient No.	Gender	Age	Cancer types	Time since diagnosis (months)
1	Female	44	Breast	9
2	Female	27	Lymph	3
3	Male	59	Chest	6
4	Female	43	Breast	10
5	Female	49	Lung	15
6	Male	72	Stomach	8
7	Female	29	Intestines	10
8	Female	52	Cervix Uteri	5
9	Female	49	Breast	3
10	Female	58	Rectum, Ovary	4
11	Male	57	Bones, Lung	15
12	Female	53	Ovary, Colon	60

*Note:* For those who have two cancer types, the second site refers to the site of cancer metastasis.

Patients were mainly recruited from a public hospital, which is the only Grade A tertiary hospital with cancer treatment in Zhangye City, Gansu Province. We also recruited three participants from Guangzhou, who came from deprived areas but were referred to a provincial hospital for joint treatment. All patients received a participant information sheet and gave consent to join the study.

### 2.2 Procedure

Ethical approval was obtained in April 2023. Semi-structured in-person interviews were conducted between April and September 2023. All interviews were conducted by the first author, who has extensive previous experience in conducting qualitative interviews and assisted by two well-trained postgraduate students.

Upon arrival, the researchers worked in collaboration with the hospital administration to establish and adhere to ethical guidelines for the study. Following approval from the hospital, researchers positioned themselves within the oncology department, covering inpatient, chemotherapy, and radiotherapy sections.

Upon expression of interest in participating in the research interviews, the researchers conducted a brief screening to ensure that patients met the inclusion criteria. In total, 50 cancer patients meeting the inclusion criteria were selected for research interviews, and fifteen agreed to participate. Three patients dropped out of the interview due to their poor health conditions.

The interviews took place in private meeting rooms provided by the collaborating hospitals in Zhangye (Gansu province) and Guangzhou (Guangdong province; Note that while all participants were residents of the deprived region, these patients were unable to access specialized oncology services locally and were referred to provincial hospitals for joint treatment). This ensured a quiet and confidential environment, free from clinical interruptions. Before beginning the formal questions, the interviewer focused on building rapport by explaining the study’s purpose in clear, simple terms, assuring confidentiality, and starting with a gentle, open-ended conversation to establish trust. Several challenges were navigated during this process. Some participants, particularly in Gansu, spoke with strong local dialects, which required a researcher familiar with the dialect to assist in ensuring accurate understanding and transcription. Furthermore, given varying educational backgrounds, interviewers occasionally needed to use simpler language and gentle prompts to help participants articulate their complex experiences. Finally, the physical toll of the illness was a key consideration; interviews were paused or rescheduled if a participant experienced pain or dizziness, prioritizing their comfort and well-being at all times.

The interviews ranged from 25 to 65 minutes, with an average duration of 35 minutes. Despite varying lengths, each interview was ended at or beyond the point of data saturation while ensuring that the participants were in a suitable health condition to be interviewed. Patients were given $12 worth of fruit vouchers, as a token of thanks to the research team. Throughout the interviews, a Dictaphone was used to record the original interview data.

### 2.3 Interview protocol

Based on Strauss and Corbin (1988) instructions for a tunnel approach to interview questions, interviews began with an open-ended question asking patients how they characterize their experience, feelings, and other essential aspects of themselves that they would like to discuss (see more details in Table II).

**Table II.** Interview question guidelines.

Open-ended questions
1. Could you please talk about the changes in your life since being diagnosed with cancer? Do you feel satisfied with your current situation?
2. Among these changes, which one(s) do you think have the most impact on your quality of life?
3. How do you cope? What was the biggest challenge you faced in seeking medical care and treatment (or in fighting the cancer)? (Use prompt questions whenever relevant and appropriate) Could you elaborate on this regarding its personal/interpersonal/community/societal aspects?
4. What are your biggest worries during the treatment process?
5. How did you come to terms with the reality of having cancer? How do you cope with negative thoughts?
6. What support have you received after being diagnosed? How have they changed, and why?
7. Do you think your cultural background and economic status have influenced your attitude toward the cancer?

### 2.4 Data analysis

The interview data were managed and analyzed using Dedoose Software (SocioCultural Research Consultants, n.d.). We employed Interpretative Phenomenological Analysis (IPA, Smith & Fieldsend, 2021), an inductive approach focused on understanding individuals' perceptions of major life events, to guide our analysis. Our process followed six key steps outlined by Smith and Fieldsend (2021): transcripts familiarization, exploratory coding, identification of emergent themes, formation of clustered themes, cross-case analysis, and final interpretation. A reflexive journal was maintained throughout to ensure the researcher's preconceptions did not unduly influence the analysis.

The process began with repeated, active reading of each transcript to achieve deep familiarization. Next, we conducted an initial exploratory coding of the transcripts on three levels: descriptive coding to note the content of patient statements, linguistic coding to examine specific language use (e.g., metaphors, pauses), and conceptual coding to begin the interpretative process by exploring underlying meanings.

From these initial codes, we identified emergent themes within each transcript. These themes represented patterns of meaning specific to each patient’s experience. These were then clustered into groups of related concepts for each case before moving to a cross-case analysis. In this stage, we looked for patterns and connections across all twelve interviews, allowing us to develop a set of superordinate themes that captured the shared experiences of the group while still honoring the individual nuances.

The final themes were established through a collaborative and iterative process of cross-checking and discussion among the research team until a unanimous agreement was reached (Nizza et al., 2021; Osborn & Smith, 1998). To ensure validity, initial thematic interpretations were also shared with several participants for their feedback.

To transparently illustrate this interpretative process, Table III provides a condensed example of the analytical progression for one specific theme. It shows how initial participant quotes (first-order analysis) were interpreted to form emergent themes (second-order analysis), which were then aggregated to create a single superordinate theme. Furthermore, a sample of a coded transcript is available in the Appendix.

**Table III.** Example of thematic development in IPA: The emergence of 'Relational Interdependent Burden.

First-Order Analysis: Participant Quote (Verbatim Text)	Second-Order Analysis: Emergent Themes (Researcher's Interpretations)	Aggregate Level: Superordinate Theme
"I make no money but spend a lot. I feel uncomfortable and guilty for my family." (Patient 8)	Sense of being a financial liability. • Guilt over the family's economic sacrifice. • Self-worth connected to economic contribution.	Relational Interdependent Burden
"My husband is disabled. I really wanted to go back to farming after the chemotherapy. I wanted to help him, but now I can't do anything." (Patient 11)	Illness prevents the fulfillment of expected family roles. • Disruption of reciprocal caregiving relationships. • Distress from perceived uselessness and dependency.	

Finally, two clinical psychology professors with expertise in qualitative research reviewed and helped refine the final set of six overarching themes to ensure conceptual clarity and rigor.

The collected data were transcribed into Chinese text. As patients from remote areas often had distinct accents, a researcher familiar with the local dialect reviewed and corrected the transcriptions to ensure accuracy. Quotations from the interviews in this article were first translated from Chinese to English by a professional translator. The bilingual research team convened to compare the original Chinese quotes with the translation. Any discrepancies or loss of emotional and cultural nuance were discussed among the team until a consensus was reached on the most accurate and authentic English rendering.

### 2.5 Researcher positionality and reflexivity

The research team recognized that our own backgrounds and perspectives are integral to the research process, particularly within an interpretative methodology like IPA. The team was composed primarily of researchers and students in clinical and health psychology, with several senior members holding PhDs from Western universities (UK) and others having backgrounds in cognitive neuroscience.

This composition brought a strength in understanding psychological distress and theory, but also introduced potential biases stemming from our clinical psychology backgrounds, Western academic training, and socioeconomic distance from the participants.

To address these issues, we embedded reflexivity throughout the research process. The reflexive journal mentioned previously was not just for notes, but for critically documenting personal reactions, assumptions, and emerging biases after each interview. Furthermore, regular team debriefing sessions were crucial. In these meetings, researchers with different backgrounds (e.g., clinical psychology vs. cognitive neuroscience) would challenge each other's interpretations, ensuring that themes were robustly grounded in the data rather than a single personal and/or theoretical viewpoint. This collaborative process helped to bracket our assumptions and remain open to the participants' lived realities. Finally, the member-checking process, where initial interpretations were shared with participants, served as a vital safeguard to ensure our final analysis resonated with patients’ own experiences.

## 3. Results

The main themes and subthemes identified are listed in Table IV.

**Table IV.** Themes and Subthemes.

Levels/Themes	Subthemes
Personal Challenges and Coping	(1) Financial Burden
	(2) Psychological Distress Caused by Treatment Side-effects
	(3) Identity Change
	(4) Self-acceptance
Interpersonal Challenges and Support	(1) Relational Interdependent Burden
	(2) Self-imposed Social Withdrawal
	(3) Relational Interdependence as Empowerment
	(4) Peer-patient Interaction
Marginalization in Healthcare System	(1) Marginalized from Healthcare Resources
	(2) Marginalized from Healthcare Policies

To ensure conceptual clarity, the definitions of the central theoretical constructs used in this analysis are provided in Table V below.

**Table V.** Definitions and operationalization of key constructs.

Key construct	Definition in this study	Example from participant data
Marginalization	The experience of being excluded from adequate healthcare resources and policies due to geographic location and socioeconomic status leads to disparities in care and feelings of powerlessness.	“The national healthcare policies are good, but the implementation in our areas turns out to be unsatisfactory.” (Patient 13)
Relational Interdependent Burden	The psychological distress and guilt experienced by patients stemming from the perception that their illness imposes a financial, practical, or emotional strain on their family and loved ones.	“I make no money but spend a lot. I feel uncomfortable and guilty for my family.” (Patient 8)
Relational Interdependence (as Empowerment)	The sense of strength, motivation, and resilience patients draw from their connections with family, friends, and peers fosters a determination to persevere through treatment for the sake of others.	“I am positive and proactive about receiving treatment, for my own sake, but also for the sake of my son and daughter.” (Patient 15)
Self-acceptance	A multifaceted coping mechanism characterized by three core components: (1) Cognitive Reframing: Reinterpreting the illness through culturally-embedded philosophical or folk-religious beliefs to find meaning; (2) Direct and Unquestioning Acceptance: Embracing suffering as a universal human experience and accepting the diagnosis as a reality without dwelling on 'why me?', which relates to a mindset of common humanity; and (3) Proactive Agency: Using this acceptance as a foundation to independently confront fears and actively engage in managing one's treatment and health.	“I never wondered 'why I got this cancer.' I accept it directly and have no fear or anxiety about it.” (Patient 6)

### 3.1 Personal challenges and coping

Participants talked about their experiences related to personal challenges, including financial burden, side effects after treatment and their psychological distress. To cope with those challenges, self-acceptance was highlighted by our participants.

Cancer treatment often places a large *financial burden on patients* in deprived areas (“The greatest negative consequence of this illness is that I cannot work anymore, meaning an unbearable financial burden”; Participant 9). Most of the cancer patients were struggling with the direct costs of medical care, including hospital stays, medications, and treatments, as well as indirect costs like travel expenses and lost wages. Those costs caused concerns and worries for patients, as Participant 8 shared:

“*For me, there are two most worrying issues: one is financial, even though I have health insurance, and the other is my deep concern about the future (whether I can recover or not)*.”

Beyond financial challenges, many patients experienced psychological distress from physical discomfort during cancer treatment. This distress was often due to side effects, including intense bodily pain, gastrointestinal issues, and emotional turmoil from changes in appearance.

For example, bodily pain is a predominant issue reported by patients, significantly impacting their psychological well-being. The most reported bodily pain included headaches, thigh or calf pain, back pain, omalgia, and stomachache. Patient 2 expressed heightened concern about her bodily pains during therapy, associating them with a deteriorating physical state and, consequently, experiencing intense feelings of worry:

“*My whole body ached. I was particularly worried about whether the pain would affect my organs….As soon as I was discharged from the hospital, my whole body ached.*”

Gastrointestinal distress, including nausea, vomiting, and loss of appetite, poses significant challenges for patients. Some patients are experiencing a long period of low appetite after treatment (“After getting chemotherapy here, I cannot eat anything for two weeks due to low appetite.”; Patient 12). Others experienced severe nausea with vomiting and felt extreme physical collapse during the onset of symptoms.

“*I am unable to eat for a week and vomit following radiotherapy. I vomit more than 20 times a night and feel like dying. At the same time, I am unable to stop my tears from flowing as a natural reaction*”. (Patient 1)

This debilitating symptomatology not only affects physical health but also exacerbates emotional distress, leading to feelings of helplessness and despair.

Moreover, patients find non-painful symptoms bothersome, such as hair loss, weight fluctuations, and acne, bothersome often give rise to appearance anxiety. This was a particularly acute source of distress for Patient 4, a 27-year-old hotel clerk recently diagnosed with lymphoma and undergoing chemotherapy. She described the devastating impact of this visible change on her emotional state and social life:

“*When my hair started to fall, I cried for a few days and could not accept it… it was so depressing and demoralizing that I was afraid of going outside*”. (Patient 4).

For many patients, such visible alterations caused by their treatment lead to anxiety and profound emotional upheaval and contribute to a distressing change in their self-identity concerning their social status and personal values.

Also, cancer patients perceived *self-identity change* after diagnosis concerning social status and personal values. Many patients expressed feelings of a drastic decline in their social standing due to their inability to work and their family income. The struggle to reconcile their previous roles and contributions with their current limitations highlights the internal conflict and diminished self-worth experienced by many patients. Some patients experienced a notable change in social standing and how others viewed them (“I used to be an engineer and be treated respectfully in the workplace, but now, things are different. I find it hard to accept my social status has changed”, Patient 3)

This crisis of identity was poignantly articulated by Patient 2, a 30-year-old unmarried teacher with a bachelor's degree who had to stop working due to her colon cancer treatment. She reflected on the profound loss of her professional role and its impact on her sense of self:

“*I can't live like a normal person anymore. It's a joke, but deep down, I worry that this won't go away. I can't work, and that's crucial for me…So it's really about my efficacy, my societal role as a teacher, and the concerns about my identity*”.

This statement highlights the patient's struggle with the loss of their previous societal roles and the associated worries about their place in society. The inability to maintain a normal life as before and fulfill their professional duties exacerbates feelings of diminished social status and a threat to their self-identity. Such reflections highlight the complex emotional terrain that cancer patients navigate in deprived areas, where societal expectations and personal aspirations intersect with the harsh realities of illness.

In addition to personal challenges and difficulties, *self-acceptance* was a central coping strategy, often expressed in religious and philosophical terms embedded in Chinese culture. This was evident with Patient 5, a married woman with a primary school education who was diagnosed with breast cancer**.** She framed her situation through a lens of pragmatic faith and resilience:

“*You accept whatever the Venerable Sky Lord [a folk deity] gives. It’s more useful to consider solutions than crying all the time*”.

Recognizing that suffering is a universal part of human life—a psychological experience known as common humanity (Neff, 2003)**-** leads to a kind of self-acceptance. This mindset, whether expressed positively or pessimistically, can improve a patient’s decision-making and psychological conditions. As Patient 1 noted, “If it cannot be cured, no one can escape it”.

Furthermore, participants showed varied attitudes, which represent a sense of self-acceptance, which in turn motivates patients to continue treatment and take proactive measures regarding their health.

Some emphasized the necessity of facing their fears independently, acknowledging that personal courage is essential despite the support from others (“No matter how much my family supports me, I know I must confront my fears on my own. This realization has empowered me to take control of my treatment”, Patient 4).

Some view their illness as an incident, accepting it with a mindset of common humanity that encourages proactive action. This perspective was clearly expressed by Patient 6, a 60-year-old woman living with stomach cancer for eight months. She articulated a mindset of direct and unquestioning acceptance: “I never wondered ‘why I got this cancer.’ I accept it directly and have no fear or anxiety about it”.

### 3.2 Interpersonal challenges and support

This section explores the complex interpersonal dynamics reported by patients, including relational burdens, social withdrawal, and supportive networks.

Patients frequently voiced concerns over the financial burdens their illness imposed on their families. For patients who are incapable of working, the financial burden of medical expenditure is frequently passed to their families, especially their son(s)/daughter(s). Patients’ sense of being a burden to others arises not only from financial issues but also from the fact that they have a disruptive influence on household duties and family plans (“I truly want to get well and go back farming…My husband will be very busy in the fields without me because he had a car accident previously and became disabled. Though he has a prosthetic leg, his body may break down with overloaded farming” (Patient 11). Many patients expressed feelings of guilt and anxiety over their families having to deplete savings, incur debt, or forgo other necessities to afford their treatment. This financial strain was articulated by Patient 8, a woman in late middle age, whose husband had quit his part-time job to care for her. During the interview, she could not help but cry when describing how difficult it was for her family to afford the next chemotherapy cycle. Overwhelmed with guilt, she shared: “I make no money but spend a lot. I feel uncomfortable and guilty for my family”.

The financial barrier forces some patients to forgo opportunities for improved treatment. This dilemma was highlighted by Patient 3, a 48-year-old Traditional Chinese Medicine practitioner diagnosed with colon cancer. Despite her own medical background and professional income, she explained how the cost of advanced care was prohibitively expensive for her family:

“*There is a cancer clinic in Wuwei [an underdeveloped city near the intended research hospital]. A deposit of 100,000 RMB (c.a. 14,000 USD) is required for better treatment there. How can I get 100,000 RMB? My daughter has a job, but her monthly salary of 3,000 RMB (c.a. 420 USD) is insufficient to cover our everyday costs and my treatment bills.*” (Patient 3)

In addition, the patients perceived *incapacity to fulfill family roles*. In many Chinese families, especially in deprived areas, they have clearly defined roles that contribute to the household's functioning and emotional well-being. For example, fathers always make money, and mothers always take care of domestic work. Cancer patients often felt a deep sense of loss and helplessness as their illness prevented them from fulfilling these roles, whether as breadwinners, caregivers, or active participants in daily family life. This disruption not only affected their self-esteem and identity but also added to their sense of being a burden, as other family members had to step in to take over their responsibilities. This sense of guilt was deeply intertwined with financial burdens, especially when a patient's need for care disrupted the family's income. This was the case for Patient 8, a woman in late middle age. With a son still in university, her family’s already stressed financial situation became more precarious. She described the resulting emotional toll:

“*My husband now must stay at home to look after me. Therefore, he cannot work or earn any money, while I keep spending all my savings every day. I feel painful and guilty about being a burden to my family*”.

Older cancer patients who would have needed to help their children or close relatives care for their offspring are unable to take on this responsibility because of their illness. For instance, Patient 12 was expected to look after the newborn child of her son. Unfortunately, after the cancer diagnosis, she was incapable of giving a hand, and her daughter-in-law had to quit her job for child-rearing, resulting in further financial stress (“My son has two babies after marriage, facing extreme stress. I cannot help my daughter-in-law, and thereby she quit her job, staying at home with the kids…What I can do is try to give some money to him apart from my medical treatment”).

Paradoxically, the care and love provided by family members, while essential and deeply appreciated, also contributed to patients' stress. Many patients reported feeling overwhelmed by the constant attention and care, which, although well-intentioned, served as a constant reminder of their dependency and the disruption their illness caused (“They are very kind to me, and I am deeply moved by this. But I am also afraid of burdening them. [the patient suddenly responds in a more dejected manner] After the treatment, I won’t be able to have children, and that will burden him [i.e., her husband], as well as the costs, those are significant burdens on him”, Patient 4).

Patients perceived themselves as a burden despite their past capabilities. The family's protective measures, while driven by love and concern, can paradoxically intensify a patient’s sense of helplessness and dependency. This role reversal was a source of significant emotional strain for Patient 13, a 59-year-old man from Gansu who, before his chest wall tumor diagnosis, prided himself on being highly capable. Now on leave from work and struggling with what he described as diagnosed anxiety and depression, he described how his family's over-protectiveness underscored his loss of autonomy:

“*I now rely on my family. Whether it's my wife or my daughter, they are always afraid of any mishap. I used to be very capable. My wife and daughter used to say I could do anything, but now I can't do anything because of the illness. For example, just drinking water at home, they (my wife and daughter) don't want me to move, fearing I might get burned when handling the kettle.*” (Patient 13).

This experience, where a once self-sufficient individual is prevented from performing even the simplest tasks, can lead to profound negative feelings. Such feelings, including embarrassment, shame, and self-degradation, often arise from the patient's internal perception of being different from their former, healthy self. These feelings led to *self-imposed social withdrawal*, emerging as the second central theme. Certain patients perceived themselves as different due to having cancer, leading to feelings of shame about both their illness and self. Over time, they developed a sense that others looked down on them, prompting them to proactively restrict their social interactions (“I am different from other people because I have cancer. I do not feel as close to my old friends as I used to, and I do not want to talk to them. I feel embarrassed to talk about having this illness”, Patient 11). This internalized stigma leads to a reluctance to seek social support, further isolating them and exacerbating their emotional distress.

In addition, patients noted that their illness made them more withdrawn and less inclined to communicate, which strained their relationships with friends and acquaintances. The perceived changes in these relationships contributed to their sense of isolation and loneliness. The shift from being an active member of their social circle to feeling like an outsider underscores the emotional and psychological toll of the disease (“Until now, when friends call, I don't answer. I don't reply to messages on WeChat [a Chinese instant chatting App] either. It's because I feel inferior and devastated. I haven't accepted this illness myself and can't talk about it with my friends”, Patient 13).

Coping strategies are important to the recovery and psychological health of cancer patients. In exploring how the patients cope with the negative experiences above, a central theme emerges: the transformative power of relational interdependence and information exchange. This theme encompasses how patients draw strength from supportive communities, navigate relational dynamics, and assert individual agency in their fight against cancer. Two interconnected subthemes shed light on these coping mechanisms: *relational interdependence as empowerment and peer-patient interaction.*.

Despite occasional feelings of being a burden, relational interdependence empowers patients through familial support and internal motivation. Patients derive strength from their families and loved ones, fostering a strong desire to persevere through treatment for their sake.

Several patients developed a positive attitude toward therapy when surrounded by caring and unwavering support from family members. Family support emerges as a cornerstone of patients' coping mechanisms, influencing their mental outlook and commitment to treatment. For instance, Patient 2's husband, characterized as an optimistic individual, consistently encouraged. Consequently, Patient 2 gained confidence in her treatment and harbored hopes of recovery (“My husband is very supportive. He told me that he was convinced that I would be cured. I was glad to hear that, so I felt much happier than before”).

Patients often express initial fears and uncertainties but find solace and motivation through the steadfast support of their loved ones, leading to a renewed determination to fight their illness. For instance, Patient 5 initially expressed a preference for a casual approach to treatment. When her daughter took proactive measures by accompanying her to larger cities for treatment, she was empowered by her daughter's intentions and actions (“I am touched by the company of my daughter, so I would like to return to the hospital in Xi'an to explore new opportunities. I would also like to travel to Beijing and Shanghai for a specialist session”*.*)

The familial interdependence also gives rise to a willingness to live for families. For instance, Patient 1 wished to live longer to see his lovely grandson growing up and decided to continue treatment, even though he thought of giving up because of the unbearable pains. Likewise, one patient pointed out:

“*I am positive and proactive about receiving treatment, for my own sake, but also for the sake of my son and daughter. I want to live a few more years to take care of them.*” (Patient 15)

Similarly, friendship and neighborhood play crucial roles in alleviating negative emotions, acting as a buffer in certain social relationships (“I could not accept my cancer at all when I was diagnosed, but my friends were so kind to me. Their support is the reason why I accepted my diagnosis; otherwise I would have been so depressed”, Patient 4).

In addition, the *peer-patient interactions* foster a sense of community and enhance patients' belief in their recovery. With information exchange and personal communications with peer patients, some pessimistic patients were empowered to have confidence in fighting cancer. Access to accurate information about their illness enhances patients' confidence in their recovery journey. Information empowers patients to actively participate in decision-making and treatment planning, contributing to their overall well-being (“I thought I could not survive for another few years, but I subsequently discovered that was not the case. After conversing with the patients on my ward, I learnt how to treat the disease at different stages and that it is curable”, Patient 4).

Through shared experiences and mutual support, patients not only gain a better understanding of treatment but also cultivate a stronger belief in their ability to overcome their illness. Patient 1, anxious about her appearance, was empowered by witnessing the positive attitudes of other patients in the hospital. This experience led her to believe that she, like everyone else, should adopt a positive attitude towards cancer and the changes it caused (“Although we all lost some hair as a result of treatment, I noticed that some patients did not wear hats to hide. People with bald heads walked around and chatted pleasantly. They all had such positive attitudes, that I felt compelled to be as well.”).

Exploring life appreciation, cultivating gratitude, identifying positive aspects amid life's challenges, broadening life perspectives, and experiencing feelings of encouragement and support were essential themes explored in the patients' narratives.

### 3.3 Marginalization in the health system

Most of our participants talked about their marginalized status when getting a cancer diagnosis and cancer treatment. Cancer patients in deprived areas often experience delayed diagnosis and are faced with limited medical resources during treatment. Two fundamental issues identified by our participants contributed to their marginalized status being marginalized from healthcare resources and health policy. The participants identified two core issues. First, being marginalized from healthcare resources meant local healthcare services were inequitably distributed. Second, being marginalized from health policy highlighted the insufficiency of the current national policies. The specific quotes are explored below.

Due to living in an area with limited healthcare resources, our participants felt upset by limited access to healthcare resources. They cannot afford to access better healthcare providers in developed areas, such as hospitals in provincial centers and far from their rural dwelling places. Also, they found it disappointing when seeking appropriate and timely responses from the public health insurance system and related medical aid programs or financial assistance for chronic diseases. Suffering these disparities and inequities, the non-central living patients were not merely geographically peripheralized but also marginalized in terms of medical resources and state-social support (“The national healthcare policies are good, but the implementation in our areas turns out to be unsatisfactory”, Patient 13).

Local healthcare providers in resource-constrained areas often have limited staff and lack up-to-date facilities. As a result, they are not always capable of accurately identifying early lesions, which can lead to misdiagnosis of the early symptoms. For example, Patient 7 was misdiagnosed with a stomach illness for months until a central city hospital correctly diagnosed cancer. The lack of early and accurate diagnosis exemplifies the resource limitations in deprived areas (“The doctor thought it was a stomach illness. Later, I began to have a constant fever and relied on medication to bring it down for a long time. I came to the current hospital [which is located in the capital city of the province]and found out I had cancer”, (Patient 7)

Additionally, the COVID−19 pandemic further strained healthcare services, causing treatment delays, as noted by Patient 1, who had to postpone cancer treatment due to staff shortages:

“*The shortage of staff in the oncology department as most doctors had to support the respiratory department or the infectious diseases department during the pandemic.*”

Some patients also highlighted the financial and logistical difficulties in accessing superior medical services far from their rural houses. Patient 11 experienced certain financial stress when she had to travel from the rural areas where she lived to the hospital located in the center:

“*It is so far from my farmhouse that I came here by public transportation costing me 50 RMB (c.a. 7 USD). So I have to rent a room for 30 RMB (c.a. 4 USD) per day here for chemotherapy*”. (Patient 11)

The distance between rural and urban healthcare facilities, both in financial and geographical terms, underscores the marginalization of rural patients. They are not only physically distanced from advanced medical resources but also politically and socially marginalized due to insufficient state-social support.

Financial burden is a significant issue for cancer patients who depend on national public health insurance for treatment reimbursement. Patients from deprived areas express dissatisfaction with the execution of health insurance policies, identifying delayed and inadequate reimbursements.

Patients commonly express dissatisfaction with the timeliness of insurance processes, including the speed of handling insurance applications and reimbursements (“I am dissatisfied with the government's policy [referring to the health insurance policy] since I am still under a lot of financial pressure. I think the national policy is sound, but everything has changed (and become more complicated) when it comes to provinces and cities”, Patient 3).

Additionally, the reimbursement rates for both Resident’s Health Insurance and the New Rural Insurance are often insufficient to alleviate financial burdens (“We, rural people, mainly rely on medical insurance for reimbursement when seeing a doctor. However, the reimbursement rate for New Rural Insurance is low, and certain costs are not covered”, Patient 6).

The patients articulated concerns and grievances regarding health insurance while contending with existing financial burdens in their economically deprived living conditions. Especially for those living in rural areas, they consistently conveyed a sense of "trouble" and expressed dissatisfaction with the perceived inadequacy of coverage in addressing their treatment expenses (“The reimbursement rate for the Resident’s Health Insurance is not high. I still have to pay the majority of the expenses by myself, which puts a lot of financial pressure on me”, Patient 9)*.* In comparison, non-rural citizens usually receive more adequate coverage from national health insurance. The *New Rural Insurance* (a new basic health social security system, started in 2003, targets all farmers and rural inhabitants), along with its various limitations, represents the difference between the rural and the urban, underlining the marginalized status of those cancer patients in deprived areas.

The discrepancy between urban and rural health insurance sufficiency further marginalizes rural cancer patients, emphasizing their peripheral status in terms of medical care resources and insurance support. This geographical and political marginalization highlights the challenges faced by cancer patients in deprived areas, reflecting a broader issue of inequity in healthcare access and support.

## 4. Discussion

The current study explored the lived experiences of cancer patients in deprived areas of China. Using IPA, our findings demonstrated the challenges faced by these patients, along with identifying supportive factors and coping strategies. The experiences of Chinese cancer patients in deprived areas are shaped by an intricate interplay of factors at multiple levels. These include personal challenges and coping (individual level), interpersonal challenges and support (interpersonal level), and marginalization in the health system (community/organizational levels). This multi-level structure is consistent with the Social Ecological Model (SEM). With the lens of the SEM and Self-construal Theory, we gain a comprehensive understanding of how different levels of factors integrating with culture interact to influence patients' lives.

### 4.1 Thematic integration

Our findings demonstrate how the lived experiences of cancer patients in rural China are shaped by a dynamic interplay across the levels of the Social Ecological Model. By integrating the SEM with Self-Construal Theory (SCT), we can move beyond a simple description of challenges to explain how systemic pressures are filtered through a cultural lens, creating a unique set of lived realities.

#### 4.1.1 Individual and community/policy levels

The significant financial burden of cancer patients at individual levels is exacerbated by the lack of external financial support, particularly health insurance, and sufficient healthcare resources at the community/policy level. Our findings confirm a large body of literature demonstrating that systemic issues, such as inadequate insurance coverage and limited healthcare access, exacerbate the financial burden on individual cancer patients. First, patients from rural areas specifically highlighted the low reimbursement rates (Li et al., 2020) and the exclusion of certain treatments from coverage (Fang et al., 2019). Second, these patients lived in deprived areas and encountered difficulties in accessing adequate medical facilities, which increases the risk of misdiagnosis (Coughlin, 2019; Manser & Bauerfeind, 2014; Van Der Kruk et al., 2022). Third, limited access to healthcare resources leads to scenarios where patients may have to forgo better treatment options due to financial constraints, and where patients need to pay additional traveling and living fees to receive better treatment from a healthcare center located far from their hometown, not covered by insurance. These extra costs can be devastating for individuals already struggling with financial constraints, resulting in encountering the same economic hardship as the prior research (Lu et al., 2019; Salsman et al., 2019).

As a result, Chinese cancer patients from deprived areas often find themselves on the periphery of healthcare systems, with inadequate access to medical resources and insurance support, thereby amplifying their financial burdens. All these contributed to the unwelcoming experience and perception of marginalization. The conception of marginalization encompasses various dimensions such as powerlessness, exclusion, and economic constraints, which collectively exacerbate the vulnerability of marginalized groups, including rural cancer patients (Madden et al., 2020; Rami et al., 2023).

The financial burden and marginalization of Chinese cancer patients in deprived areas can be profoundly understood through the concepts of 'creation of margins', 'living between cultures,' and 'creation of vulnerabilities' according to the findings of Baah and his colleagues (2019). For Chinese cancer patients in deprived areas, the creation of margins is evident in the stark disparities in the duality between the deprived areas they inhabit and the more developed urban centers where advanced medical care is available (Xiao et al., 2020; Zhao et al., 2020). The movement between rural and urban settings not only highlights the inequalities but also imposes additional financial and psychological burdens. This journey not only imposes financial and physical strains but also places patients in a liminal space where they must reconcile their rural identities with the demands and expectations of urban medical environments. The cultural dissonance can be profound, as rural patients may encounter language barriers (local dialect vs. Mandarin), different social norms, and a healthcare system that does not cater to their specific needs. Moreover, the exposure to advanced healthcare facilities juxtaposed with the inadequate provisions in their home areas reinforces feelings of marginalization and neglect (Zou et al., 2020). These negative financial, social, and structural factors contribute to increased vulnerability and decreased survivability among the cancer patients (Baah et al., 2019; Rami et al., 2023)

While marginalization can often lead to negative psychological outcomes, it can also paradoxically foster a sense of self-acceptance in some patients, as they are forced to confront and adapt to their circumstances with a reliance on internal resources and culturally ingrained coping mechanisms. In line with previous research (Chen et al., 2017), patients’ self-acceptance developed over time. This process often involves reframing their cancer-related suffering within the larger (folk)religious narratives (e.g., the illness as divine-given) or normalizing the suffering as a universal human experience, helping reconcile their marginalized experiences with broader existential realities and fostering a sense of solidarity with others who suffer, regardless of socio-economic status, heightened by marginalization. This self-acceptance is a paradox. It is partly a passive resignation to their circumstances and partly a proactive stance that empowers them to take control. This dual mindset enables them to navigate their cancer journey and marginalized status with a sense of agency.

Therefore, our study not only confirms the systemic issues indicated by previous research, but its findings extend this by illuminating a paradoxical link between systemic powerlessness and individual agency. Faced with a marginalizing healthcare system that offered little external control, patients were often forced to turn inward. This process fostered a unique form of self-acceptance, rooted in a mindset of common humanity, which became a critical tool for reclaiming a sense of agency. Thus, a policy-level failure does not simply create hardship; it shapes the very nature of the individual's internal coping strategies

#### 4.1.2 Individual and interpersonal levels

The inadequacies in healthcare policies and insurance coverage are systemic issues and translate into financial crises for patients and their families. In the context of marginalization, patients often experience a heightened sense of interdependence, where their identity and well-being are closely tied to their social and familial networks.

The financial and emotional support from family members becomes crucial in navigating the healthcare system and coping with the illness. This finding resonates with the concept of an interdependent self-construal (Giacomin et al., 2017; Li et al., 2024), often described as a significant element in many East Asian cultural contexts**,** where an individual's identity is deeply interwoven with a larger social network, seeing him/herself as part of a larger social network and prioritizing group harmony and familial obligations over personal desires (Li et al., 2024; Li et al., 2020). However, in deprived areas, the families of patients often bear the brunt of caregiving responsibilities. This can strain interpersonal relationships, as family members struggle to provide adequate care amidst their own financial and emotional challenges. More importantly, the findings in this study suggested that relational interdependence, especially familial support, while crucial, also contributes to this complex dynamic. The tendency for patients to express deep concern about the financial and emotional strain on their families appears to be strongly informed by a cultural emphasis on relational interdependence (Cross et al., 2020; Markus et al., 1991). For instance, many patients expressed guilt and anxiety over their families’ depleting savings and incurring debt to afford cancer treatments, highlighting their deep sense of responsibility towards their loved ones. On the other hand, the relational interdependence that characterizes the patients' social context means that familial care, though supportive, can also amplify their sense of being a burden. Patients often feel overwhelmed by the constant attention and care, which, while well-intentioned, serve as persistent reminders of their dependency and the disruption their illness causes to their family’s daily lives. Moreover, this intense care also led to feelings of burden and loss of autonomy. Patients often felt that their illness imposed significant emotional and practical burdens on their loved ones, which in turn heightened their sense of helplessness and dependency. This dichotomy highlights the double-edged nature of relational interdependence. While familial support provides a crucial emotional lifeline, it simultaneously reinforces the patients' sense of being a burden, contributing to their emotional distress (cf. J.-J. Chen et al., 2021).

Patients’ strong connections to family and community, which exacerbate feelings of shame and burden, may also lead to self-imposed social withdrawal. Patients often internalize their illness as a source of stigma, fearing the negative impact their suffering might have on their loved ones. While previous research has established a link between illness-related shame and voluntary social withdrawal (Little et al., 2022; Wang et al., 2015), our findings indicate a culturally specific mechanism driving this phenomenon. In the context of an interdependent self-construal, the shame patients experienced was not merely personal but deeply relational—a profound guilt over being a burden to the family collective. This drives them to avoid social interactions, not just to hide their own vulnerability, but to actively shield their friends and family from distress. Thus, while social isolation is a well-documented challenge for cancer patients across diverse age groups (Fox et al., 2023; Pahl et al., 2021), our research specifies that the motivation for this withdrawal can be rooted in a protective, albeit isolating, desire to maintain social harmony and minimize disruption to the lives of loved ones.

Nevertheless, the study found complex dynamics between individual and interpersonal experiences. The vital role of support networks is the main coping strategy of cancer patients at the interpersonal level (Chen et al., 2021). This encapsulates the transformative power of relational interdependence and information exchange, highlighting how patients draw strength from their intimate relations and communities. Despite the relational interdependent burdens, familial interdependence instils a strong desire in patients to persevere through treatment for the sake of their loved ones (Van Der Kruk et al., 2022). In addition, several patients reported a positive shift in their attitude toward therapy when surrounded by the unwavering support of their families and encouraged to foster a sense of hope and determination. This finding is consistent with previous research despite the unique cultural context. Studies suggest that intimate relationships with family and friends allow emotional encouragement to reach patients’ innermost feelings. This, in turn, fosters their resilience and improves their quality of life (Chen et al., 2021; Sibeoni et al., 2018; Usta, 2012).

Furthermore, engaging with fellow patients and exchanging experiences also forms a crucial aspect of the support network. Peer-patient interactions foster a sense of community, enhance belief in recovery, and provide practical insights into managing the illness. Access to accurate information about their illness further empowers patients. Information exchange among peers helps demystify the disease, reduce anxiety, and bolster confidence in the recovery journey. This aligns with previous research suggesting the importance of the cancer knowledge and diagnostic acumen of patients in deprived areas of China (Wang et al., 2022).

The individual-interpersonal complex dynamics also appeared regarding the coping mechanism. On the one hand, while self-acceptance is central to patients’ coping mindset on the individual level, the burden patients feel they impose on their loved ones may challenge their sense of self-acceptance. On the other hand, family support was found to provide emotional strength that bolsters the patient's self-acceptance, particularly evident in the accounts of patients who, despite their initial fears and doubts, find renewed determination to fight their illness through the encouragement of their family members (Chen et al., 2021). More importantly, interactions with other patients bridge the individual and interdependent coping strategies. Sharing experiences with peers who are undergoing similar struggles can enhance a patient’s self-acceptance by normalizing their experiences and reducing feelings of isolation. This communal aspect of coping can transform self-acceptance from a solitary act into a shared journey, where patients draw strength from each other’s acceptance and resilience (Chen et al., 2021).

#### 4.1.3 Interpersonal and community/policy levels

Regarding the interaction between the interpersonal and community/policy levels, the systemic inadequacies in healthcare resources and insurance coverage create significant financial and psychological pressures on cancer patients and their families (Chu et al., 2021). Here, we see how the frameworks of SEM and Self-construal Theory become analytically generative. The policy-level stressor (inadequate insurance) is not experienced in a vacuum; it is mediated through the cultural lens of an interdependent self-construal.

Specifically, an interdependent self-construal transforms an external, systemic pressure into an internalized, interpersonal crisis. In our study, the financial strain of cancer care was not experienced merely as a logistical problem but became a source of profound guilt and a sense of being a burden on one's family. This process was poignantly articulated by Patient 8, who, overwhelmed with guilt, shared: ‘I make no money but spent a lot. I feel uncomfortable and guilty for my family’. As this quote exemplifies, the cultural framework of interdependence amplifies the policy-level failure, causing it to manifest as relational distress. These pressures can therefore strain familial relationships, as the responsibility of caregiving often falls on family members who are already stretched thin by their own economic and emotional challenges. The lack of external support from community resources or government policies means that families must rely heavily on their limited resources, which can exacerbate feelings of helplessness and frustration.

In addition, the marginalized status may foster feelings of helplessness and despair, contributing to self-imposed social withdrawal as patients cope with the emotional and psychological impacts of their exclusion. The lack of adequate support systems in marginalized communities means that patients often feel they have nowhere to turn for emotional support, reinforcing their decision to isolate themselves. Marginalization, through the creation of physical, economic, and cultural barriers, leads to increased isolation. This isolation, in turn, exacerbates the marginalization as patients withdraw from the few available support networks, reducing their access to potentially beneficial social and community resources (Su et al., 2022; Zhao et al., 2022).

Conversely, the quality of interpersonal relationships can significantly influence how patients and their families navigate the broader systemic barriers. Strong familial support can mitigate some of the negative impacts of inadequate healthcare policies by providing emotional and practical assistance that helps patients cope with their illnesses. Moreover, interpersonal relationships can serve as a buffer against the feelings of marginalization that arise from systemic exclusion. This resilience is particularly evident when families work together to navigate the complexities of the healthcare system, such as negotiating with providers or finding alternative treatment options that may be more affordable or accessible (Su et al., 2022).

However, the reliance on interpersonal support also highlights the vulnerability of patients who lack strong family or community ties. In the absence of robust interpersonal networks, patients may find themselves particularly vulnerable to the systemic challenges of the healthcare system. Without the emotional and practical support of loved ones, these patients may experience increased isolation, making it more difficult for them to advocate for their needs or seek out the care they require (Chen et al., 2021; Usta, 2012).

While existing research notes that systemic healthcare inadequacies strain family resources, our unique integration of SEM and SCT reveals the cultural mechanism driving this process. A policy failure, like insufficient insurance, is not merely a logistical challenge for the family; through the lens of an interdependent self-construal, it is transformed into an internalized, interpersonal crisis of guilt and relational distress.

#### 4.1.4 An integrative framework of multidimensional lived experiences of cancer patients in Chinese-deprived areas

**Figure 1.**
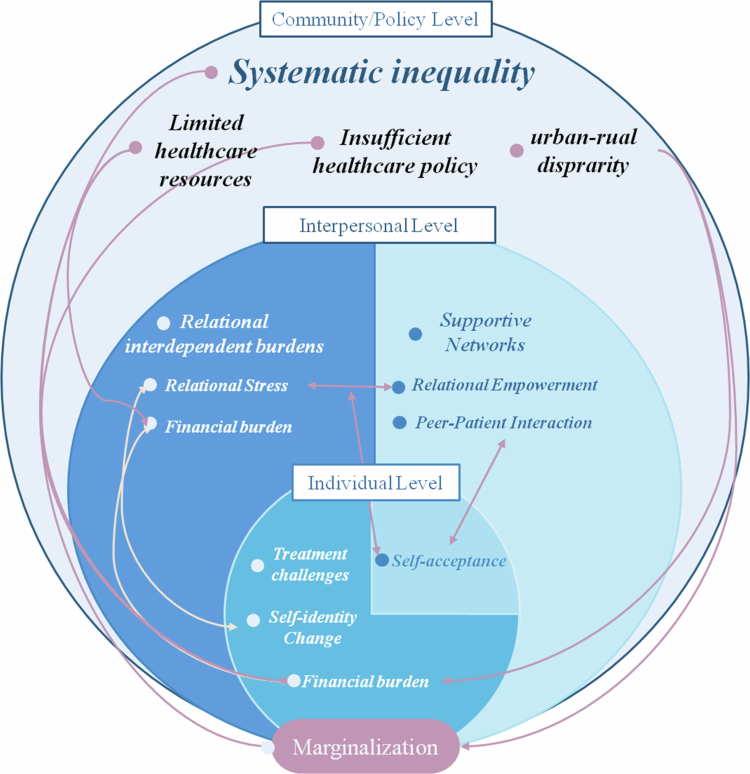
The integrative framework of the lived experiences based on the Social Ecological Model.

The insights gained from this study highlight the multidimensionality of the lived experiences of cancer patients in deprived areas of China, illustrating how these experiences are shaped by a complex interplay of individual, interpersonal, and community/policy factors (see Figure 1). Systematic inequality was found to be the fundamental determinant of the overall condition of the deprived patients, which was characterized by marginalization. Specifically, the limited healthcare resources, insufficient health insurance policy, and general urban-rural disparity resulting from systematic inequality as such, contributed to unbearable financial burdens, which exacerbated the psychological stress of both individual patients and their families. Facing these challenges, self-acceptance emerged as the fundamental coping mechanism on a personal level. Despite its complex dynamics concerning relational interdependence (which is experienced as a source of both burden and empowerment), it works well with the peer-patient interaction as another main coping strategy on an interpersonal level. The supportive networks appeared as a buffer zone between the helplessness and suffering of vulnerable patients and the overall unsupportive community.

Most of the themes found in this study underscore the complex, multifaceted nature of coping strategies among cancer patients. Relational interdependence, peer-patient interactions, and self-acceptance all play crucial roles in shaping patients' experiences and responses to their illnesses. A nuanced application of SEM and Self-construal Theory, mindful of their limitations, can enhance our understanding of these dynamics, ultimately contributing to better support and care for cancer patients.

In summary, the key themes of this study should not be viewed as discrete categories but as deeply interconnected components of a single, integrated experience. Systemic marginalization acts as the foundational stressor, which is then interpreted through the cultural lens of relational interdependence, leading to both profound burdens and sources of empowerment. In response to this complex reality, a form of self-acceptance rooted in a mindset of common humanity emerges as a critical, albeit paradoxical, coping mechanism that allows patients to navigate their journey with a sense of agency.

### 4.2 Integrating the social ecological model and self-construal theory in exploring lived experiences of patients with severe illness

Understanding the multifaceted lived experiences of cancer patients in deprived areas requires a theoretical framework that can account for both broad systemic influences and deeply ingrained cultural dynamics. The Social Ecological Model (SEM) offers valuable frameworks for mapping these various levels of influences—from the individual to the societal. However, SEM on its own falls short in explaining *why* individuals within a specific culture navigate these levels in the way they do. This is where Self-construal Theory (SCT) becomes essential. SCT provides the necessary cultural lens, explaining how values like relational interdependence shape a person's identity and choices. In essence, while SEM draws the map of a patient's world, SCT explains how their cultural background teaches them to read that map.

The primary strength of SEM lies in its multi-level approach. Yet its common depiction as a series of nested, hierarchical layers can constrain the interpretation of cultural fluidity and the complex, non-linear ways in which patients experience and respond to their illness. Our findings suggest that a patient’s experience does not always follow this rigid structure. For instance, consider the theme of self-acceptance. While this is fundamentally an individual-level strategy for coping with the illness itself, its role appears to be amplified by the context of systemic powerlessness. Faced with a healthcare system that made them feel peripheral and marginalized, patients were often forced to turn inward to the one area where they could reclaim agency: their own mindset. In this way, an individual-level coping strategy (self-acceptance) becomes a crucial response to the powerlessness felt from systemic marginalization at the policy level. This fluid, bi-directional link between the individual and policy levels defies a strictly hierarchical interpretation. Furthermore, an overemphasis on the individual at the center of the model can obscure the profound impact of systemic barriers, such as inadequate healthcare access and support, which are beyond the individual’s control. This perspective can be limiting for marginalized cancer patients. It may neglect the need for systemic changes at the community or policy level. Such changes are necessary to address the root causes of marginalization and improve their overall well-being. Indeed, this focus has led scholars to propose alternative "inside-out" approaches to the SEM that center policy and environment (Golden et al., 2015).

To address these limitations, particularly the model's underestimation of cultural context, we integrate Self-construal Theory (SCT). SCT provides the crucial cultural lens needed to understand *why* the SEM’s levels are so deeply intertwined and fluid for this patient population. For the individuals with a strong interdependent self-construal, the ‘self’ is not a discrete entity but is defined by and inseparable from the relationships and systems it inhabits. From this perspective, a policy-level failure, like insufficient insurance coverage, can be simultaneously felt as both a personal and interpersonal failing. Conversely, an individual act of self-acceptance becomes an act of redefining one's role and value within the family and community.

However, we are mindful that SCT itself has limitations. Critics argue that its emphasis on a strict divide between independence and interdependence can oversimplify the complexity of cultural identities​ (Giacomin et al., 2017; Li et al., 2024). Studies have shown that self-construals are not mutually exclusive and can coexist within individuals (Cross et al., 2010), and scholars like Matsumoto, (1999) have challenged this binary classification, advocating for a more fluid understanding of self-identity.

Moreover, there are mixed findings among the studies based on the Self-construal Theory. For instance, while some studies show that interdependent self-construal is associated with better social support and emotional well-being, others suggest that it may also lead to increased stress due to heightened sensitivity to social harmony and expectations (Li et al., 2024). Li et al. (2006) suggested the possibility that China has emerged as a middle ground between collectivistic and individualistic features. These mixed findings highlight the need for a more nuanced approach to studying self-construal in diverse cultural and clinical settings.

Indeed, our findings reflect this complexity. Patients’ experiences were not purely interdependent; they often leveraged familial support to exercise their own agency and independence. For instance, Patient 5, who was initially passive about her cancer care, was empowered by her daughter’s support to proactively seek better treatment options, expressing a new desire to travel to specialist hospitals in Beijing and Shanghai. This highlights that self-construal is not a fixed trait but a dynamic construct, especially in the face of a severe illness. Furthermore, a life-altering event like a cancer diagnosis can itself be a catalyst for shifts in self-perception, meaning that a person's self-construal is not merely a pre-existing cultural script but is actively negotiated and can evolve throughout the illness journey. Therefore, while our findings highlight a strong tendency toward interdependent concerns, this should be understood as a prevalent pattern within our sample rather than a uniform trait of all patients in this context.

Ultimately, it is the synthesis of SEM and SCT that offers the most robust and nuanced explanatory framework. The two theories are reciprocally strengthening. SEM provides the architectural blueprint of influence—mapping out the critical policy, community, interpersonal, and individual levels. SCT, in turn, explains the cultural dynamics of how patients navigate that architecture—how they internalize systemic pressures and mobilize personal and relational resources in response. This integrated approach allows us to move beyond simple categorization to a more profound understanding of how culture shapes lived reality, creating a framework that is both structurally comprehensive and culturally sensitive.

### 4.3 The gendered dimensions of illness and deprivation

It is critical to acknowledge that with a sample comprised of 75% women, our thematic findings are inevitably shaped by gendered experiences. For instance, the profound distress related to bodily changes, such as hair loss and its impact on public appearance, may reflect heightened societal pressures on women's bodies and identities. Furthermore, one patient's fear that an inability to have children would burden her husband highlights how the illness intersects with deeply ingrained gender roles surrounding reproduction and family life.

Similarly, the theme of relational interdependent burden appears strongly gendered. Many narratives of guilt and anxiety stemmed from an inability to fulfill traditional caregiving roles,such as managing household duties, farming alongside a husband, or looking after grandchildren,which are disproportionately assigned to women in many cultural contexts, particularly in deprived rural areas. Therefore, the lived experience of cancer for many women in this study is not just about the illness itself, but about the intersection of illness, deprivation, and gender, where the physical and psychological toll is amplified by the inability to perform expected social roles.

### 4.4 Implications

The study highlights the urgent need for policy reforms that address the healthcare disparities in deprived areas. The marginalization of cancer patients in these regions from healthcare resources and policies calls for a more inclusive approach to healthcare planning and resource allocation. Policies must be developed to ensure equitable access to cancer treatment and support services, particularly in economically disadvantaged areas. Specifically, policy-makers may consider reforming health insurance policies (particularly the New Rural Insurance) to better address the significant non-medical costs associated with cancer care. Alleviating the burdens of travel, accommodation, and lost wages—costs which our findings identify as major sources of financial hardship—would be a critical step toward more equitable care for rural patients.

On the interpersonal level, given the relational burdens and social isolation experienced by these patients, there is a clear need for interventions that foster social support and community engagement. Healthcare providers should be trained to recognize and address the psychological and social dimensions of cancer care, ensuring that patients have access to comprehensive support that includes emotional counseling, peer support groups, and community-based resources. For instance, Hospitals serving rural populations should pilot locally adapted patient navigation programs. Navigators—specialized nurses or social workers—could provide one-on-one support to help patients manage the logistical complexities of their care, from scheduling appointments and deciphering insurance paperwork to connecting them with financial aid and peer support groups. This would directly address the "trouble" and dissatisfaction patients feel when interacting with the healthcare system.

The study's findings also emphasize the need for culturally sensitive care that takes into account the predominant interdependent self-construal among Chinese cancer patients. By integrating these frameworks, this study can analyze not only the multi-levelled systemic barriers patients face (via SEM) but also the cultural lens through which they interpret and respond to these challenges (via SCT), providing a holistic answer to our research question. In this regard, oncology staff should receive training that is directly informed by the cultural dynamics revealed in this study. This training should focus on how to engage family members in a way that respects their supportive role while centering the patient’s autonomy, thereby minimizing the patient’s sense of being a burden. Also, oncology staff should be equipped to recognize signs of self-imposed social withdrawal and proactively connect patients to hospital-based or community peer support networks, which this study found to be a powerful source of empowerment and coping.

Finally, the study highlights the need for ongoing research to better understand the complex interplay between socioeconomic status, cultural context, and health outcomes in cancer care. Further qualitative and quantitative studies are necessary to explore the long-term effects of cancer treatment on patients in deprived areas and to evaluate the effectiveness of different intervention strategies. More importantly, future studies should move toward designing and evaluating the effectiveness of the interventions proposed here. This gives rise to specific research questions, such as: ‘To what extent does a culturally-informed patient navigation program reduce psychological distress for rural cancer patients?’ and ‘What is the impact of family-centered communication training on patient-reported feelings of burden?’ This could include pilot studies on the efficacy of patient navigation programs or randomized controlled trials on the impact of culturally-informed training on patient satisfaction and treatment adherence. Such research is essential to build an evidence base for scaling up effective solutions for this vulnerable population.

### 4.5 Limitations

It is crucial to recognize several limitations in the current study. Firstly, the recruitment of predominantly female participants (75%) is a significant limitation. While this study provides deep insights into the illness experience, these findings are likely dominated by narratives reflecting gendered social roles and pressures faced by women. As our discussion in Section 4.3 noted, themes of bodily image, reproductive concerns, and disruptions to caregiving are deeply intertwined with female identity in this cultural context. Consequently, this study lacks a diverse gender perspective. Future research should purposefully recruit and explore the lived experiences of male cancer patients in similar deprived settings to understand how gender may mediate the experience of illness and deprivation differently for men, who might face alternative societal pressures (e.g., stoicism, the role of the breadwinner).

Secondly, recruiting patients from economically deprived areas with lower educational backgrounds may have restricted their ability to provide detailed information during interviews. Future investigations should incorporate quantitative measures, such as scales or experimental research methodologies, to validate the themes identified in the current study. Integrating rigorous empirical approaches will not only bolster the reliability and validity of the identified themes but also contribute to a more comprehensive understanding of the phenomena under examination.

## 5. Conclusion

This study offers a comprehensive exploration of the lived experiences of cancer patients in deprived areas of China, shedding light on the profound and multifaceted challenges they face. By integrating the Social Ecological Model (SEM) and Self-construal Theory, the research captures the dynamic interplay between individual, interpersonal, community, and policy-level factors that shape the illness experience of these patients.

The findings underscore the significant impact of socio-economic deprivation on the psychological and emotional well-being of cancer patients, revealing how financial burdens, treatment challenges, and self-imposed social withdrawal are deeply intertwined with cultural and social norms. The study also highlights the critical role of support networks and the complexities of self-acceptance in coping with the illness.

Moreover, this research emphasizes the necessity for culturally sensitive and context-specific interventions that address the unique needs of cancer patients in socio-economically disadvantaged regions.

By integrating the SEM with Self-construal Theory, the study provides a nuanced understanding of how cultural values and socioeconomic status influence the lived experiences of cancer patients, offering valuable insights for future healthcare policies and support programs. This synthesis reveals two key insights. First, it exposes the ‘double-edged sword’ of interdependence: the same familial relationships that provide crucial empowerment also create profound emotional burdens and guilt, often leading to self-imposed social withdrawal. Second, it shows how systemic powerlessness can foster a unique form of self-acceptance, rooted in a mindset of common humanity, which functions as a critical tool for patients to reclaim a sense of agency. Our central contribution, therefore, is to demonstrate how macro-level policy failures manifest as intimate, interpersonal crises.

In conclusion, this study contributes a culturally-attuned and structurally-aware framework for understanding the suffering of marginalized patient populations. We argue that effective policies and interventions must address not only the objective, systemic barriers patients face but also the cultural meanings they attach to their illness and relationships. The findings call for ongoing research and intervention efforts that consider the broader socio-cultural and economic realities faced by these patients, ultimately aiming to improve their quality of life and psychological well-being.

## Ethical statement

Ethical approval for this study was obtained from the Human Research Ethics Committee of the School of Psychology at South China Normal University (Ethical approval number: SCNU-PSY−152). All participants provided written informed consent before enrollment in the study.

## Data Availability

For noncommercial and research purposes, the corresponding author may be contacted to obtain the study's data. Due to privacy and ethical concerns, the data is not made public.

## Appendix

### Sample of Coded Interview Excerpt

**Table:**

Transcript excerpt	Researcher’s comments and codes
"I make no money but spend a lot. I feel uncomfortable and guilty for my family. My husband has to work and also take care of me. It's too much for him. I just feel like I'm a burden." (Patient 8)	**Descriptive Code:** • Patient expresses feeling guilty about the financial cost of treatment and the practical strain on her husband. **Linguistic Code:** • Use of strong emotional language: "uncomfortable," "guilty," "burden." • Juxtaposition of "make no money" and "spent a lot" highlights the perceived economic imbalance. **Conceptual/Interpretative Code:** • The patient connects the financial cost of her illness directly to her interpersonal value, seeing herself as a "burden." Her identity as a contributor is inverted, causing significant psychological distress. This reflects a deep sense of responsibility within the family unit. → **Emergent Theme:** Financial strain as an interpersonal failing. → **Emergent Theme:** Guilt over disrupting family roles.
“The national healthcare policies are good, but the implementation in our areas turns out to be unsatisfactory. I think the national policy is sound, but everything has changed (and become more complicated) when it comes to provinces and cities.” (Patient 13)	**Descriptive Code:** • Patient describes a disconnect between the positive intent of national policies and the negative reality of their local execution. **Linguistic Code:** • Use of contrasting structure: "policies are good, **but** implementation. unsatisfactory." • This highlights a perceived gap or failure in the system. • Phrasing like "everything has changed" suggests a feeling of being let down by local bureaucracy. **Conceptual/Interpretative Code:** • The patient experiences a sense of powerlessness; they can see the "good" policy from a distance but cannot access its benefits due to their location. • This points to a systemic failure that leaves the individual feeling peripheral to the state's intended care. → **Emergent Theme:** Disconnect between policy and practice. → **Emergent Theme:** Feeling of geographic and political exclusion.
